# The Association Between the Dietary Inflammatory Index, Dietary Pattern, and Hypertension Among Residents in the Xinjiang Region

**DOI:** 10.3390/nu17010165

**Published:** 2025-01-01

**Authors:** Min Wang, Jiali Liao, Hao Wang, Lu Deng, Tingyu Zhang, Heng Guo, Xin Qian, Rulin Ma

**Affiliations:** 1 Department of Preventive Medicine, Shihezi University, Shihezi 832000, China; wangmin1@stu.shzu.edu.cn (M.W.); liaojiali@stu.shzu.edu.cn (J.L.); wanghao@stu.shzu.edu.cn (H.W.); denglu@stu.shzu.edu.cn (L.D.); zhangtingyu@stu.shzu.edu.cn (T.Z.); guoheng@shzu.edu.cn (H.G.); qianxin@shzu.edu.cn (X.Q.); 2 Key Laboratory for Prevention and Control of Emerging Infectious Diseases and Public Health Security, The Xinjiang Production and Construction Corps, Shihezi 832000, China

**Keywords:** hypertension, dietary inflammatory index, dietary patterns, factor analysis, logistic  regression model

## Abstract

**Background**: Diet and inflammation are both associated with hypertension. We aimed to investigate the relationship between the dietary inflammation index (DII), dietary patterns, and the risk of hypertension among Xinjiang residents. **Methods**: A total of 930 residents aged 20–80 from Shihezi and Tumushuk were selected as participants using a stratified whole cluster random sampling method. General demographic information, dietary data, and physical examination results were collected from the participants and DII scores were calculated. Restricted cubic spline was used to analyze the dose–response relationship between the DII and the risk of hypertension. LASSO regression was used to screen dietary factors associated with hypertension. Factor analysis was used to extract dietary patterns. Finally, logistic regression modeling was used to analyze the association between the DII, dietary patterns, and the risk of hypertension. **Results**: The DII was linearly and positively associated with the risk of developing hypertension. Logistic regression analysis showed that the prevalence of hypertension was 2.23 (95% CI: 1.53, 3.23) and 3.29 (95% CI: 2.26, 4.79) in the T2 and T3 groups, respectively, compared with the T1 group. Riboflavin and folate were associated with the risk of hypertension. In the vegetable–egg dietary pattern, the risk of hypertension was reduced by 33%, 39%, and 37% in groups Q2, Q3, and Q4, respectively, compared with group Q1 (Q2: OR = 0.67, 95% CI: 0.45, 0.99; Q3: OR = 0.61, 95% CI: 0.41, 0.92; Q4: OR = 0.63, 95% CI: 0.42, 0.96). **Conclusions**: The higher the DII score, the higher the risk of hypertension among residents of Xinjiang. In addition, vegetable–egg dietary patterns can reduce the risk of hypertension. Therefore, local residents should be scientifically instructed to increase their intake of vegetables and eggs.

## 1. Introduction

Hypertension is a clinical condition defined by elevated arterial blood pressure within the circulatory system, which may be associated with functional or structural damage to vital organs such as the heart, brain, and kidneys [1]. Reports indicate that in 2019, China represented 18.85% of the approximately 1.3 billion individuals diagnosed with hypertension globally, and the incidence of this condition is increasing, particularly among younger populations [2]. Hypertension represents a significant risk factor for cardiovascular disease, adversely affecting the quality of life within the population and imposing a considerable economic burden. Various risk factors contribute to the development of hypertension, including age, tobacco use, alcohol consumption, dietary habits, and exposure to air pollution [3]. An unhealthy diet contributes to the accumulation of adipose tissue and increased blood glucose levels, leading to inflammation and oxidative stress. This physiological condition can precipitate endothelial dysfunction and atherosclerosis, thereby hastening the development of hypertension [4]. Research indicates that consuming red meat, refined grains, and sugary beverages correlates with elevated levels of inflammation. In contrast, consuming vegetables, fruits, whole grains, and dietary fiber is linked to reduced inflammatory responses [5].

The Dietary Inflammatory Index (DII), a novel instrument developed by Shivappa et al., is designed to evaluate the inflammatory potential of individuals [6]. The influence of diet-induced inflammation on the onset and progression of various diseases is studied. Research indicates that an increase in DII scores correlates with elevated levels of interleukin-6 (IL-6), tumor necrosis factor-alpha (TNF-α), and C-reactive protein (CRP), as well as an increased risk of coronary heart disease development [7,8,9]. The dietary pattern refers to the quantity, proportions, types or combinations of different foods, beverages, and nutrients in the diet and the frequency with which they are habitually consumed [10]. Prior research has predominantly concentrated on individual foods or nutrients, often overlooking the interactions between food items [11,12,13,14]. Identifying dietary patterns facilitates a more comprehensive investigation into the association between diet and disease, highlighting the potential influence of various dietary patterns on health outcomes. A meta-analysis encompassing 13 distinct dietary patterns indicated that the cessation of hypertensive dietary therapy was the most effective approach for mitigating the risk of hypertension. Furthermore, the Mediterranean diet and low-carbohydrate dietary patterns demonstrated the next most favorable effects [15]. Xinjiang, situated in the northwest region of China, is characterized by a distinctive geographical environment and dietary habits. The prevalence of hypertension in this area stands at 31.2% [16], surpassing the national average of 27.5% [2]. Consequently, this study aims to examine the relationship between the dietary inflammatory index, dietary patterns, and the risk of hypertension in Xinjiang. The findings aim to offer valuable insights into the prevention and management of hypertension within this region.

## 2. Materials and Methods

### 2.1. Research Objects

**Figure 1:**
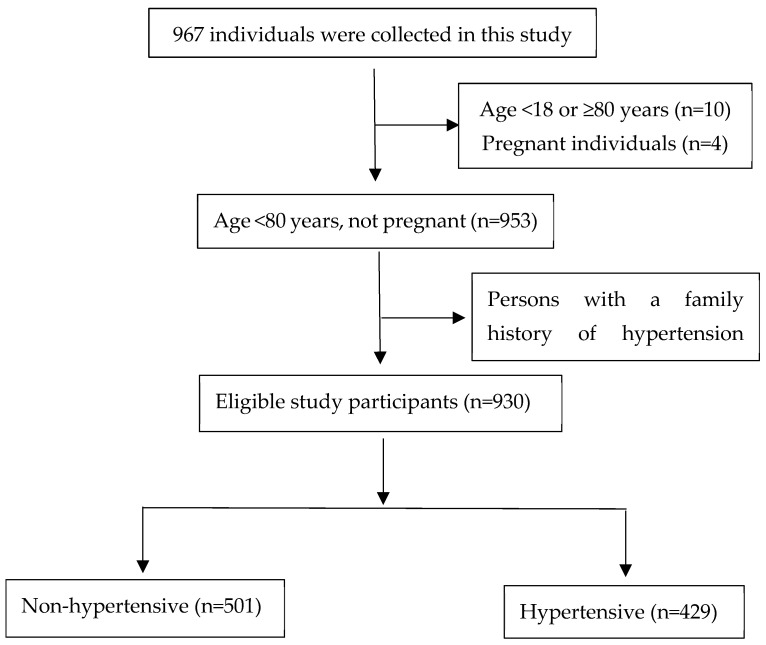
Flow chart of the study population.

In this research, a stratified whole-group sampling method was employed to identify participants who had resided in the cities of Shihezi and Tumushuke for a minimum duration of one year and were between the ages of 20 and 80. A total of 967 people were collected as follows: 4 pregnant individuals, 10 individuals aged <20 years or >80 years, and 23 individuals with a family history of the disease were excluded, resulting in the inclusion of 930 research subjects, as seen in Figure 1. This research received approval from the Ethics Review Committee of the First Affiliated Hospital of Shihezi University, under Ethics Review Number KJ2023-410-01. All participants in the study provided their informed consent by signing a consent form. The inclusion criteria for the study were as follows: (1) Participants must be aged between 20 and 80 years; (2) Participants must have resided at the study location for a minimum of one year; (3) Participants must agree to partake in the survey and sign the informed consent form. The exclusion criteria included the following: (1) Males with an energy intake of less than 500 kcal/day or exceeding 8000 kcal/day, and females with an energy intake of less than 500 kcal/day or exceeding 5000 kcal/day; (2) Pregnant individuals and those with mental disorders that impair communication; (3) Individuals suffering from chronic wasting diseases, such as tumors; (4) Individuals with a family history of hypertension [17].

### 2.2. Questionnaire Survey

The questionnaire included general demographic information and dietary information. The demographic data encompassed a range of variables, including gender, age, race, literacy level, smoking behaviors, alcohol consumption, and medical history. A semi-quantitative food-frequency questionnaire was employed to evaluate the dietary practices of the study population and examine the frequency of food consumption and the amount of food intake per occasion over the preceding year. The questionnaire comprised categories such as staple foods, livestock and meat, aquatic products, vegetables, fruits, potatoes, and dairy products.

### 2.3. Physical Examination

Height, weight, waist circumference, and blood pressure were assessed by uniformly trained professionals utilizing calibrated equipment by standardized protocols. Participants in the study were instructed to remove their shoes, hats, and heavy clothing before measuring height and weight. After five minutes of seated rest, blood pressure was measured in the right arm using an Omron arm sphygmomanometer (Dalian, China). Each subject underwent two consecutive measurements at five-minute intervals, and the average of these two readings was recorded.

### 2.4. Dietary Inflammation Index

A semi-quantitative food-frequency questionnaire assessed the frequency and quantity of food consumption over the past year within the study population. The specific calculations conducted are as follows: (1) The mean daily dietary intake for each participant was determined through a dietary survey, calculated as follows: mean daily dietary intake = (grams of food per intake × frequency of intake). (2) The Z-score for each dietary component within the study population was computed using the formula that follows: Z = (daily intake of the dietary component—global per capita daily intake of the dietary component)/standard deviation of global per capita daily intake. (3) Z-scores were then converted into percentiles to mitigate the impact of right skewness. The resulting percentile values were multiplied by “2” and subsequently reduced by “1” to achieve a symmetrical distribution centered around “0”, with boundaries ranging from −1 to 1. (4) The adjusted percentiles were multiplied by the corresponding inflammatory effect index for each dietary component to derive the Dietary Inflammatory Index (DII) for individual dietary ingredients. (5) The DII values for all dietary components were aggregated to calculate a total individual DII score. In this study, DII scores were derived based on 29 dietary nutrients, which include energy, protein, carbohydrates, dietary fiber, cholesterol, total fat, saturated fatty acids, monounsaturated fatty acids, polyunsaturated fatty acids, *n*-3 fatty acids, *n*-6 fatty acids, trans-fatty acids, carotenoids, vitamin A, thiamine, riboflavin, vitamin C, vitamin D, vitamin E, vitamin B_6_, vitamin B_12_, folate, niacin, iron, magnesium, selenium, zinc, anthocyanins, and isoflavones. The study participants were divided into three groups according to the tertiles of the DII score: T1 (anti-inflammatory diet group), T2 (intermediate diet group) and T3 (pro-inflammatory diet group).

### 2.5. Dietary Patterns

The full dietary information of the study population, including staple food categories such as livestock and meat, aquatic products, vegetables, fruits, potatoes, mushrooms, dairy products, eggs, and nuts, was included in the factor analysis, and KMO test results and Bartlett’s sphericity were performed to determine whether the study was suitable for factor analysis. The male factors with eigenvalues greater than 1 and a cumulative variance contribution greater than 66.0% were selected for inclusion in the study. All foods or food groups with an absolute value of the factor loading matrix >0.50 were retained in the model, and the dietary patterns were named according to factor interpretability and the characteristics of the foods contained in the food pattern.

### 2.6. Relevant Definitions

(1) Hypertension is defined as individuals with a prior diagnosis of hypertension who are currently receiving antihypertensive treatment, or those exhibiting a systolic blood pressure (SBP) ≥ 140 mmHg (1 mmHg = 0.133 kPa) and/or diastolic blood pressure (DBP) ≥ 90 mmHg, in the absence of antihypertensive medication [18]. (2) Diabetes mellitus is characterized as a condition affecting individuals who have received a definitive diagnosis of diabetes mellitus and are undergoing pharmacological treatment to regulate their blood glucose levels or have a fasting blood glucose (FBG) ≥ 7.0 mmol/L [19]. (3) Smoking is defined as either exceeding a total of 100 cigarettes consumed or engaging in continuous smoking for six months [20]. (4) Alcohol consumption is defined as the intake of alcoholic beverages, such as beer and spirits, occurring two or more times per month [21].

### 2.7. Quality Control

Before the administration of the questionnaire survey, designated personnel received specialized training to ensure a comprehensive understanding of the survey’s objectives and content. The investigator provided a detailed explanation of the questionnaire’s purpose and content before the survey commenced. Physical examinations were carried out by uniformly trained professionals who accurately measured height, weight, waist circumference, and blood pressure utilizing precise equipment by standardized protocols. Upon the completion of the survey, the investigators reviewed the questionnaires for completeness and engaged with respondents promptly to address any inaccuracies or errors in the responses, thereby enhancing the quality of the data collected.

### 2.8. Statistical Analysis

Data analysis was conducted utilizing R version 4.3.1 and SPSS version 25.0. The study population was categorized into three groups based on the baseline DII scores. Normally distributed variables are presented as (*x* ± *s*), while non-normally distributed variables are reported as [*M* (*P*_25_, *P*_75_)]. Categorical data are expressed as frequencies or proportions. The Wilcoxon rank-sum and Kruskal–Wallis tests were employed to assess differences in continuous variables between groups. In contrast, the chi-squared (χ^2^) test was utilized to compare categorical variables across groups. Restricted cubic spline (RCS) analysis was employed to investigate the dose–response relationship between the DII and the risk of hypertension. Additionally, logistic regression modeling was utilized to assess the association between the DII, dietary patterns, and the likelihood of developing hypertension. Furthermore, a LASSO regression analysis, conducted using the “glmnet” package in R, was implemented to identify the significant dietary factors correlated with hypertension. The “plot” package was utilized to develop a nomogram illustrating the dietary factors that exhibited a strong correlation with the risk of hypertension. The discriminative ability of a nomogram chart model to identify disease risk was assessed using the receiver operator characteristics curve (ROC). Factor analysis was used to extract the research population’s dietary patterns. The difference was statistically significant at *p* < 0.05.

## 3. Results

### 3.1. Characteristics of the Study Population

This study finally comprised 930 participants, with a mean age of 56.0 (44.0, 65.0) and a weighted median DII score of 0.28 (−0.88, 1.37) for the entire cohort. When comparing the hypertensive group to the non-hypertensive group, it was observed that the hypertensive individuals were older, had lower levels of education, and exhibited higher rates of smoking and alcohol consumption (*p* < 0.05). Table 1 provides detailed information about the baseline characteristics of research participants according to their status as hypertensive. According to the Dietary Guidelines for Chinese Residents (2016), the consumption levels of aquatic products, vegetables, fruits, tubers, dairy, and eggs among individuals without hypertension were significantly higher than those observed in the hypertensive group (*p* < 0.05), as in Table 2. The DII scores were higher in the hypertensive than the non-hypertensive population (0.75 vs. −0.26, *p* < 0.001). A thorough examination of the dietary components showed that, compared to those without hypertension, those in the hypertensive group consumed less of each nutrient. Table 3 shows that except for trans fatty acids, carbohydrates, cholesterol, and vitamin E, the difference in nutrient intake between the two groups was statistically significant (*p* < 0.05). The participants in the study were classified into three groups based on the tertiles of DII scores: T1 (<−0.49), T2 (−0.49, 0.98), and T3 (≥0.98). It was observed that individuals with higher DII scores exhibited a lower intake of nutrients compared to those in the T1 group. The analysis of the various DII subgroups revealed statistically significant differences (*p* < 0.001) in the intake of all nutrients, except for anthocyanins, across the three groups, as detailed in Table 4.

### 3.2. Association of the DII with the Risk of Developing Hypertension

**Figure 2:**
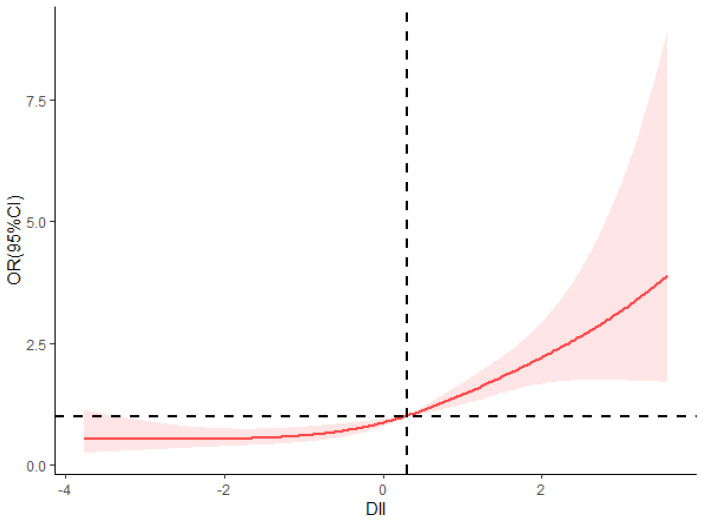
Restricted cubic spline curve of DII and the risk of hypertension.

After controlling for the confounding variables, the likelihood of developing hypertension tended to rise with increasing DII scores (P_trend_ < 0.001). Logistic regression analysis indicated that the prevalence of hypertension was 2.23 (95% CI: 1.53–3.23) and 3.29 (95% CI: 2.26–4.79) in the T2 and T3 groups, respectively, in comparison to the T1 group, as detailed in Table 5. The results from the restricted cubic spline analysis demonstrated that a continuous increase in the DII was linearly and positively correlated with the prevalence of hypertension, even after adjusting for variables such as sex, age, literacy, ethnicity, smoking, alcohol consumption, diabetes mellitus, CVD, and BMI (P_total trend_ < 0.001, P_nonlinear_ = 0.104). Furthermore, the risk of developing hypertension escalated with higher DII scores when the DII exceeded 0.26, as in Figure 2.

### 3.3. Lasso Regression and Nomogram

**Figure 3:**
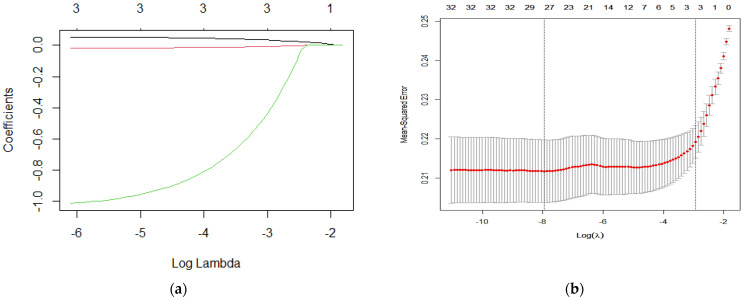
LASSO regression screening for key dietary factors associated with hypertension. (**a**) LASSO regression coefficient plots. (**b**) Cross-validation plots. LASSO regression, minimum absolute contraction, and selection operator.

**Figure 4:**
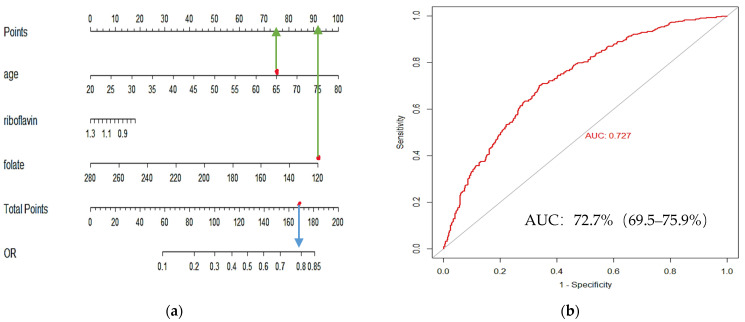
Development and validation of a predictive model for hypertension risk. (**a**) Column nomogram of key dietary factors associated with hypertension identified by LASSO regression. The green lines indicate the individual scores for each variable, and the blue lines indicate the risk of developing hypertension corresponding to the total score when the individual scores for both variables are added together. (**b**) ROC curves evaluating the predictive ability of the column-line graph model for hypertension. LASSO: least absolute shrinkage, and selection operator; ROC: receiver operator characteristic curve.

Key hypertension-related dietary factors were screened using LASSO regression, which incorporated 29 nutritional components and three demographic variables (sex, age, and literacy)—selecting the penalty parameter λ based on a 10-fold cross-validation. In this study, the algorithm was performed for 1000 iterations to ensure accuracy, and the critical and appropriate number of variables was obtained when λ was 0.048, in Figure 3b. There were three variables including age, riboflavin, and folate, as seen in Figure 3a. The variables screened through the LASSO regression culminated in a nomogram in which two variables (age and folate) contributed statistically significantly to the model, as seen in Figure 4a. ROC analysis was performed with variables contributing to the model, such as age and folic acid, to obtain ROC curves. The area under the working curve (AUC) of the subjects’ characteristics of the model was 72.7% (95% CI: 65.9–75.9%). Therefore, the model demonstrates good accuracy, as seen in Figure 4b.

### 3.4. Construction of Dietary Patterns

All dietary intake data from the study participants were incorporated, and dietary patterns were derived through factor analysis. The Kaiser–Meyer–Olkin (KMO) test results yielded a value of 0.649. In contrast, the Bartlett’s test of sphericity indicated a significance level of *p* < 0.001, confirming the appropriateness of factor analysis for this study. The variance maximization rotation method was employed to identify common factors with eigenvalues exceeding 1. Food items or groups exhibiting an absolute value in the factor loading matrix greater than 0.50 were retained in the model. The dietary patterns were subsequently designated based on the interpretability of the factors and the characteristics of the foods included in each pattern, as detailed in Table 6.

### 3.5. Logistic Regression of Different Dietary Patterns and Prevalence of Hypertension

Four distinct dietary patterns were identified through factor analysis: the livestock meat–nuts–dairy, aquatic products–mushrooms–tubers, vegetables–eggs, and staple-foods patterns. Factor scores for each dietary pattern were grouped by quartiles (Q1–Q4 groups). A logistic regression analysis, which accounted for potential confounding variables, revealed that the vegetable–egg dietary pattern significantly decreased the risk of hypertension among Xinjiang residents by 33% in the Q2 group (OR = 0.67, 95% CI: 0.45, 0.99), 39% in Q3 group (OR = 0.61, 95% CI: 0.41, 0.92), and 37% in the Q4 group (OR = 0.61, 95% CI: 0.41, 0.92),as detailed in Table 7.

## 4. Discussion

The research was undertaken to examine the relationship between the DII, dietary patterns, and the risk of hypertension among adult residents of Xinjiang. The findings indicated that the average DII score was significantly higher in individuals with hypertension compared to those without. Furthermore, the study established a linear positive correlation between the DII score and the likelihood of developing hypertension, even after controlling for potential confounding variables. The DII score in this analysis was derived from 29 dietary components, and two specific components—riboflavin and folate—were identified as having a strong association with the risk of hypertension. In addition, a nomogram was developed by integrating folate levels with age, and the resulting model demonstrated good predictive capabilities for hypertension. For example: If a patient is 65 years old and has a single score of 75 and a daily folic acid intake of 120 ug, the single score is 92, and the total score is the sum of the two single scores is 167. The blue arrow from 167 gives a risk of hypertension of 0.78, which is relatively high for this patient.

Hypertension is a clinical condition defined by an increased systolic and diastolic blood pressure (SBP/DBP), with inflammation being a significant contributing factor to its development [22]. In the research conducted by Aboukhater et al. [23], a significant correlation was identified between reactive oxygen species (ROS), interleukin-1 (IL-1), IL-6, TNF-α, and hypertension. Furthermore, the study by Malesza et al. [24] revealed that the consumption of animal fats, processed meats, and refined grains, characteristic of a Western diet, contributes to an imbalance in gut microbiota, dysfunction of the intestinal barrier, heightened intestinal permeability, and the systemic circulation of bacterial metabolites, thereby exacerbating inflammatory conditions. Additionally, a high-fat diet has been associated with increased levels of pro-inflammatory cytokines, including CRP, IL-6, TNF-α, and interferon, which collectively promote oxidative stress and an inflammatory state, consequently elevating the risk of chronic diseases [25]. The consumption of whole grains, nuts, and aquatic products has been shown to exert antioxidant effects, regulate fat metabolism, and diminish insulin resistance within the body, thereby lowering the risk of non-communicable diseases [26]. The DII is an innovative instrument developed to evaluate the correlation between dietary habits and inflammation. In this research, we conducted a comparative analysis of DII scores and their 29 constituent components between hypertensive and non-hypertensive populations in Xinjiang. Our findings indicate that the average DII was significantly higher in hypertensive individuals compared to their non-hypertensive counterparts, corroborating the results reported by Zhou et al. [27]. The current research identified a linear positive correlation between the DII score and the risk of hypertension within the study population (p-nonlinear = 0.104). This finding is consistent with the studies conducted by Vissers et al. [28] and Neufcourt et al. [29]. Furthermore, the risk of hypertension was found to be significantly elevated when the DII exceeded 0.26.

Diet plays a significant role in the modulation of inflammation. Research indicates that vegetables and fruits are abundant in essential nutrients, including dietary fiber, minerals, antioxidants, and vitamins, which may contribute to the attenuation of inflammatory responses and the mitigation of hypertension risk [30,31,32]. Additionally, the consumption of *n*-3 fatty acids, folate, and monounsaturated fatty acids has been associated with a decreased likelihood of developing hypertension [33,34,35]. In this study, we found that the intake of vegetables, fruits, and nuts was greater in the non-hypertensive population than in the hypertensive population, and the intake of 29 dietary components was greater than in the hypertensive population. Meanwhile, we identified riboflavin and folate as important nutritional components associated with the risk of developing hypertension. McNulty et al. [36] showed that riboflavin plays an important role in energy metabolism, cellular antioxidant potential, and metabolism of other micronutrients. When riboflavin intake is reduced, it increases the risk of hypertension. Folate plays an important role in DNA methylation, amino acid, and lipid homeostasis by regulating one-carbon metabolism, and folate deficiency leads to hyperhomocysteinemia, which increases the risk of cardiovascular disease and cancer [37]. Zhang et al. [38] have indicated that adequate folate intake is associated with a reduced risk of hypertension and a decreased disease burden, which aligns with the results of our study. However, the key factors associated with hypertension screened in this study were different from those of Zhou et al. [27]. This may be because Xinjiang is a multi-ethnic region with different dietary patterns among ethnic groups. Residents are more inclined to consume animal offal, nuts, and dairy products, while the intake of fruits and vegetables is relatively low, leading to vitamin deficiencies in the body and increasing the risk of inflammatory responses and the development of hypertension. In this research, a nomogram was ultimately developed utilizing two out of the three identified variables. The variables, namely age and folate, contributed statistically significantly to the predictive model. The nomogram established in this study exhibited strong predictive capabilities for hypertension.

This study presents both strengths and limitations. Among its strengths are the following: (i) the investigation of the potential positive correlation between the DII and the risk of hypertension utilizing restricted cubic spline curves (RCS); (ii) the application of LASSO regression to identify the primary dietary factors significantly associated with the prevalence of hypertension, along with the development of a nomogram to enhance hypertension prediction; (iii) four different dietary patterns were modeled in the population of Xinjiang through factor analysis, and it was concluded that a vegetable and egg dietary pattern reduced the risk of hypertension in this population. However, there are some limitations of this study: (i) the relatively small sample size of this study and the fact that all participants were from Xinjiang suggests geographic constraints; therefore, expanding the geographic scope and sample size is essential for conducting a more comprehensive study; (ii) this study relied on a food-frequency questionnaire to collect data on participants’ past dietary habits, which may introduce subjective bias and recall bias; (iii) the current research is a cross-sectional study and is unable to establish a causal relationship between the DII and the risk of developing hypertension.

## 5. Conclusions

In summary, our findings indicate a significant linear positive correlation between the DII and hypertension, whereby elevated DII scores correspond to an increased risk of developing hypertension. Furthermore, adherence to a vegetables–eggs dietary pattern was associated with a 37 percent reduction in hypertension risk. In this research, a nomogram model developed from essential dietary factors identified through LASSO regression demonstrated good predictive capabilities for hypertension. Consequently, residents must receive evidence-based guidance to adopt a healthier dietary regimen, which should emphasize the consumption of anti-inflammatory foods—such as low-sugar fruits and fiber-rich items—while minimizing the intake of pro-inflammatory foods, including fried items and processed meats. This approach aims to mitigate the risk of hypertension and alleviate the healthcare burden within the community.

## Figures and Tables

**Table 1:** General information about the study population.

Variables	Total Population (*n* = 930)	Non-Hypertensive Population (*n* = 501)	Hypertensive Population (*n* = 429)	χ^2^ Value	*p*-Value
Gender				5.78	<0.05 *
Male	388 (41.7)	191 (49.2)	197 (50.8)		
Female	542 (58.3)	310 (57.2)	232 (42.8)		
Age group				88.14	<0.001 ***
20~43	218 (23.4)	163 (74.8)	55 (25.2)		
44~55	222 (23.9)	132 (59.5)	90 (40.5)		
56~64	249 (26.8)	129 (51.8)	120 (48.2)		
65~80	241 (25.9)	77 (31.9)	164 (68.1)		
Literacy level				15.20	<0.01 **
Primary School and below	450 (48.4)	215 (47.8)	235 (52.2)		
Junior High School	297 (31.9)	184 (61.9)	113 (38.1)		
Middle/High School	115 (12.4)	62 (53.9)	53 (46.1)		
College/Undergraduate	68 (7.3)	40 (58.8)	28 (41.2)		
Nation				2.34	0.13
The Uygurs	587 (63.1)	305 (52.0)	282 (48.0)		
The Han nationality	343 (36.9)	196 (57.1)	147 (42.9)		
Smoking				7.97	<0.01 **
Yes	160 (17.2)	70 (43.7)	90 (56.3)		
No	770 (82.8)	431 (56.0)	339 (44.0)		
Drinking				5.66	<0.05 *
Yes	125 (13.4)	55 (44.0)	70 (56.0)		
No	805 (86.6)	446 (55.4)	359 (44.6)		
Diabetes				5.36	0.02 *
Yes	50 (5.4)	19 (38.0)	31 (62.0)		
No	880 (94.6)	482 (54.8)	398 (45.2)		
CVD				25.34	<0.001 ***
Yes	95 (10.2)	28 (29.5)	67 (70.5)		
No	835 (89.8)	473 (56.6)	362 (43.4)		
BMI (kg/m^2^)				33.72	<0.001 ***
<18.5	10 (1.1)	8 (80.0)	2 (20.0)		
18.5~	243 (26.1)	159 (65.4)	84 (34.6)		
24.0~	337 (36.2)	189 (56.1)	148 (43.9)		
≥28.0	340 (36.6)	145 (42.6)	195 (57.4)		

Note: Variables are presented as a number with a percent (categorical). CVD, Cardiovascular disease; BMI, Body Mass Index. *** *p* < 0.001, ** *p* < 0.01, * *p* < 0.05.

**Table 2:** Comparison of food intake by food groups in the study population (g/day).

Variables	Non-Hypertensive Population (*n* = 501)	Hypertensive Population (*n* = 429)	*z* Value	*p*-Value
Staple foods	449.6 (432.8, 467.2)	447.2 (429.8, 467.3)	1.11	0.27
Livestock and meat	142.7 (99.9, 170.8)	141.1 (101.4, 162.3)	0.99	0.32
Aquatic products	4.2 (2.1, 8.1)	3.2 (0.9, 7.5)	3.40	0.001 **
Vegetables	242.8 (220.8, 270.7)	230.1 (206.0, 257.9)	5.08	<0.001 ***
Fruits	220.9 (207.1, 237.7)	212.6 (199.5, 224.7)	6.55	<0.001 ***
Tubers	31.7 (27.3, 36.3)	29.8 (26.3, 34.8)	3.54	<0.001 ***
Mushrooms	9.7 (6.7, 15.2)	8.0 (6.0, 13.2)	3.90	<0.001 ***
Dairy	64.0 (48.0, 97.8)	60.0 (43.0, 85.0)	2.03	0.04 *
Eggs	28.8 (24.6, 35.5)	27.1 (21.7, 33.2)	3.79	<0.001 ***
Nuts	21.8 (16.0, 27.8)	21.6 (16.7, 26.5)	0.67	0.50

Note: Variables are expressed as the median (interquartile range). *** *p* < 0.001, ** *p* < 0.01, * *p* < 0.05.

**Table 3:** Dietary nutrient intake of the study population.

Variables	Non-Hypertensive Population (*n* = 501)	Hypertensive Population (*n* = 429)	*t*/*z* Value	*p*-Value
DII	−0.26 (−1.21, 0.81)	0.75 (−0.21, 1.94)	9.39	<0.001 ***
Energy (kcal)	2060 ± 110	2030 ± 120	3.76	<0.001 ***
Protein (g)	106 (100, 112)	102 (95, 111)	4.84	<0.001 ***
Total fat (g)	68.9 (63.0, 75.5)	65.9 (60.3, 73.3)	3.65	<0.001 ***
Saturated fatty acids (g)	12.0 (11.2, 13.1)	11.5 (10.4, 12.7)	4.79	<0.001 ***
Monounsaturated fatty acids (g)	10.1 (9.4, 11.2)	9.8 (9.0, 10.9)	3.91	<0.001 ***
Polyunsaturated fatty acids (g)	6.1 (5.6, 6.8)	5.9 (5.3, 6.7)	4.01	<0.001 ***
*n*-3 fatty acids (g)	1.4 (1.3, 1.5)	1.4 (1.3, 1.5)	4.26	<0.001 ***
*n*-6 fatty acids (g)	11.4 (10.7, 12.1)	10.9 (10.3, 11.8)	4.79	<0.001 ***
Trans fatty acids (g)	1.7 (0.1, 1.9)	1.6 (0.1, 1.8)	1.13	0.26
Carbohydrates (g)	363.1 ± 22.4	360.1 ± 22.2	1.83	0.07
Dietary fiber (g)	12.9 (12.2, 13.7)	12.5 (11.6, 13.5)	5.52	<0.001 ***
Cholesterol (mg)	312 (198, 376)	302 (205, 355)	1.73	0.08
Vitamin A (μg RE)	827 (767, 871)	797 (750, 846)	4.26	<0.001 ***
Carotene (μg)	2950 (2610, 3270)	2810 (2530, 3140)	2.87	<0.01 **
Thiamine (mg)	1.3 ± 0.1	1.2 ± 0.1	4.63	<0.001 ***
Riboflavin (mg)	1.0 (1.0, 1.1)	1.0 (0.9, 1.1)	6.06	<0.001 ***
Magnesium (mg)	376 (121, 403)	368 (123, 391)	2.09	<0.05 *
Iron (mg)	27.6 ± 2.1	26.9 ± 2.3	4.12	<0.001 ***
Zinc (mg)	14.4 (13.4, 15.2)	13.9 (13.2, 14.8)	3.70	<0.001 ***
Selenium (μg)	51.1 (48.6, 54.4)	49.4 (46.0, 53.3)	5.11	<0.001 ***
Vitamin C (mg)	72.8 (65.3, 100.2)	67.0 (60.4, 93.7)	4.85	<0.001 ***
Vitamin D (μg)	1.6 (1.4, 4.6)	1.5 (1.3, 4.4)	3.52	<0.001 ***
Vitamin E (mg)	13.6 (7.2, 14.6)	13.5 (7.3, 14.3)	1.56	0.12
Vitamin B_6_ (mg)	0.9 (0.7, 1.1)	0.8 (0.7, 1.1)	3.59	<0.01 **
Vitamin B_12_ (μg)	4.7 (4.4, 5.1)	4.4 (4.1, 4.8)	5.68	<0.001 ***
Folic acid (μg)	187 (174, 201)	175 (162, 194)	5.90	<0.001 ***
Niacin (mg)	19.9 (18.5, 21.5)	18.9 (17.4, 21.0)	4.51	<0.001 ***
Anthocyanin (mg)	20.5 (17.1, 25.1)	19.2 (16.1, 23.5)	2.42	<0.05 *
Isoflavonoids (mg)	0.1 (0.0, 0.7)	0.0 (0.0, 0.6)	2.77	<0.01 **

Note: Variables are expressed as mean ± standard deviation (normal distribution), and the median (interquartile range) (non-normal distribution). RE: Retinol Equivalents. *** *p* < 0.001, ** *p* < 0.01, * *p* < 0.05.

**Table 4:** Comparison of dietary nutrients among different DII populations.

Variables	T_1_ Group	T_2_ Group	T_3_ Group	*H* Value	*p*-Value
Energy (kcal)	2100 (2040, 2180)	2040 (1960, 2120)	1970 (1900, 2040)	214	<0.001 ***
Protein (g)	108 (103, 112)	106 (101, 114)	97 (93, 104)	206	<0.001 ***
Total fat (g)	68.9 (63.6, 74.8)	64.1 (58.7, 70.9)	58.2 (54.1, 63.4)	203	<0.001 ***
Saturated fatty acids (g)	12.1 (11.4, 13.0)	12.3 (11.3, 13.6)	10.7 (10.0, 11.9)	150	<0.001 ***
Monounsaturated fatty acids (g)	10.6 (9.9, 11.4)	10.1 (9.4, 11.2)	9.2 (8.5, 9.9)	185	<0.001 ***
Polyunsaturated fatty acids (g)	6.5 (6.1, 7.2)	6.1 (5.6, 6.9)	5.4 (5.1, 5.9)	233	<0.001 ***
*n*-3 fatty acids (g)	1.5 (1.4, 1.6)	1.4 (1.3, 1.5)	1.3 (1.2, 1.4)	222	<0.001 ***
*n*-6 fatty acids (g)	11.7 (11.2, 12.4)	11.2 (10.7, 12.0)	10.4 (10.0, 11.0)	244	<0.001 ***
Trans fatty acids (g)	1.8 (0.1, 1.9)	1.5 (0.1, 1.8)	1.6 (0.1, 1.7)	126	<0.001 ***
Carbohydrates (g)	320 (360, 390)	310 (340, 370)	310 (340, 370)	99	<0.001 ***
Dietary fiber (g)	13.1 (12.7, 13.7)	12.9 (12.2, 14.0)	11.7 (11.3, 12.5)	233	<0.001 ***
Cholesterol (mg)	360 (240, 390)	220 (180, 340)	300 (210, 340)	97	<0.001 ***
Vitamin A (μg RE)	830 (780, 880)	820 (760, 870)	780 (740, 820)	80	<0.001 ***
Carotene (μg)	3040 (2680, 3420)	2850 (2530, 3170)	2750 (2480, 3080)	49	<0.001 ***
Thiamine (mg)	1.3 (1.2, 1.3)	1.3 (1.2, 1.3)	1.2 (1.2, 1.2)	202	<0.001 ***
Riboflavin (mg)	1.0 (1.0, 1.1)	1.0 (1.0, 1.1)	0.9 (0.90, 1.0)	220	<0.001 ***
Magnesium (mg)	405 (132, 416)	126 (117, 388)	366 (130, 378)	194	<0.001 ***
Iron (mg)	28.5 (27.4.29.6)	27.2 (26.1, 28.4)	25.6 (14.4, 26.6)	284	<0.001 ***
Zinc (mg)	14.8 (14.1, 15.6)	14.1 (13.3, 15.0)	13.6 (12.9, 14.4)	121	<0.001 ***
Selenium (μg)	51.7 (50.0, 54.0)	51.2 (48.6, 55.8)	46.5 (44.7, 49.2)	214	<0.001 ***
Vitamin C (mg)	70.2 (65.1, 86.3)	90.0 (66.7, 104.6)	63.1 (58.8, 84.3)	125	<0.001 ***
Vitamin D (μg)	1.5 (1.4, 3.2)	4.1 (1.5, 4.9)	1.4 (1.2, 3.8)	118	<0.001 ***
Vitamin E (mg)	14.9 (8.2, 15.4)	7.6 (6.9, 14.1)	13.3 (7.6, 13.8)	202	<0.001 ***
Vitamin B_6_ (mg)	0.8 (0.7, 1.0)	1.0 (0.8, 1.2)	0.7 (0.6, 0.9)	114	<0.001 ***
Vitamin B_12_ (μg)	4.6 (4.35, 4.98)	4.7 (4.4, 5.1)	4.4 (4.1, 4.7)	72	<0.001 ***
Folic Acid (μg)	186 (176, 196)	188.5 (173.9, 208.1)	165.8 (157.6, 184.8)	154	<0.001 ***
Niacin (mg)	20.1 (19.0, 21.1)	20.3 (18.6, 22.5)	17.7 (16.8, 19.5)	176	<0.001 ***
Anthocyanin (mg)	19.7 (16.6, 24.4)	20.4 (16.6, 24.7)	19.1 (16.2, 23.3)	5	0.08
Isoflavonoids (mg)	0.1 (0.0, 0.4)	0.1 (0.0, 0.7)	0.0 (0.0, 0.5)	95	<0.001 ***

Note: Variables are expressed as the median (interquartile range). RE: Retinol Equivalents. *** *p* < 0.001.

**Table 5:** Logistic regression of the DII and the risk of developing hypertension.

DII Grouping	Model 1				Model 2		Model 3		
	OR	95% CI	*p*-Value	OR	95% CI	*p*-Value	OR	95% CI	*p*-Value
T_1_	1.00	-	-	1.00	-	-	1.00	-	-
T_2_	2.15	(1.54, 3.00)	<0.001 ***	2.26	(1.58, 3.24)	<0.001 ***	2.23	(1.53, 3.23)	<0.001 ***
T_3_	4.38	(3.12, 6.14)	<0.001 ***	3.70	(2.60, 5.27)	<0.001 ***	3.29	(2.26, 4.79)	<0.001 ***
P_trend_ value			<0.001 ***			<0.001 ***			<0.001 ***

Note: Data are presented as OR and 95% CI. The study subjects were divided into three groups, T1, T2, and T3, according to the DII scores, with the T1 group as the reference group, with no adjustment for Model 1. Model 2 was adjusted for gender, age, literacy, and ethnicity, and Model 3 was further adjusted for smoking, alcohol, diabetes mellitus, CVD, and BMI based on Model 2. DII: Dietary Inflammatory Index; CVD: Cardiovascular disease; BMI: Body Mass Index; OR: odds ratio; CI: confidence interval. *** *p* < 0.001.

**Table 6:** Construction of dietary patterns.

Factor 1	Factor Load	Factor 2	Factor Load	Factor 3	Factor Load	Factor 4	Factor Load
Livestock-meat	**0.86**	Aquatic products	**0.77**	Vegetables	**0.89**	Staple foods	**0.93**
Nuts	**0.83**	Mushrooms	**0.74**	Eggs	**0.72**	Tubers	**0.33**
tubers	**0.33**	Tubers	**0.51**	Dairy	**0.26**	Dairy	**0.30**
Vegetables	**0.03**	Dairy	**0.32**	Mushrooms	**0.22**	Livestock-meat	**0.12**
Staple foods	**0.001**	Fruits	**0.20**	Fruits	**0.13**	Nuts	**0.12**
Aquatic products	**−0.21**	Vegetables	**0.11**	Livestock-meat	**0.08**	Fruits	**0.05**
Eggs	**−0.27**	Nuts	**0.08**	Tubers	**0.06**	Vegetables	**0.02**
Mushrooms	**−0.39**	Eggs	**0.05**	Aquatic products	**0.02**	Eggs	**−0.01**
Fruits	**−0.47**	Staple foods	**−0.12**	Staple foods	**−0.02**	Mushrooms	**−0.12**
Dairy	**−0.61**	Livestock-meat	**−0.17**	Nuts	**−0.18**	Aquatic products	**−0.14**
Eigenvalue	3.06		1.37		1.14		1.03
Variance contribution %	30.62		13.65		11.44		10.30
Cumulative contribution %	30.62		44.27		55.71		66.01

Note: Factor 1: Livestock–meat–nuts–dairy dietary pattern; Factor 2: Aquatic products–mushrooms–tubers dietary pattern; Factor 3: Vegetables–eggs dietary pattern; Factor 4: Staple foods dietary pattern. Bold: Factor loadings are measures of strength between the original variable and the corresponding factor in factor analysis.

**Table 7:** Logistic regression of dietary patterns and the prevalence of hypertension.

Dietary Patterns	Model 1	Model 2			Model 3	
	OR (95% CI)	*p*-Value	OR (95% CI)	*p*-Value	OR (95% CI)	*p*-Value
Livestock–meat–nut–dairy pattern						
Q_1_	1.00		1.00		1.00	
Q_2_	1.39 (0.96, 2.01)	0.08	1.18 (0.73, 1.90)	0.50	1.24 (0.76, 2.00)	0.39
Q_3_	1.37 (0.94, 1.97)	0.10	1.01 (0.54, 1.90)	0.98	1.04 (0.54, 1.97)	0.92
Q_4_	0.998 (0.69, 1.45)	0.99	0.83 (0.44, 1.58)	0.57	1.05 (0.54, 2.03)	0.88
Aquatic product–mushroom–tuber pattern						
Q_1_	1.00		1.00		1.00	
Q_2_	0.73 (0.51, 1.05)	0.09	0.72 (0.49, 1.06)	0.10	0.83 (0.55, 1.25)	0.37
Q_3_	0.52 (0.36, 0.76)	<0.001 ***	0.59 (0.39, 0.87)	0.009 **	0.71 (0.47, 1.07)	0.10
Q_4_	0.58 (0.40, 0.84)	0.004 **	0.65 (0.43, 0.99)	0.039 **	0.82 (0.53, 1.25)	0.35
Vegetable–egg pattern						
Q_1_	1.00		1.00		1.00	
Q_2_	0.61 (0.42, 0.88)	0.008 **	0.67 (0.46, 0.99)	0.04 *	0.67 (0.45, 0.99)	0.04 *
Q_3_	0.49 (0.34, 0.71)	<0.001 ***	0.60 (0.40, 0.88)	0.003 **	0.61 (0.41, 0.92)	0.02 *
Q_4_	0.42 (0.29, 0.61)	<0.001 ***	0.59 (0.39, 0.88)	0.01 *	0.63 (0.42, 0.96)	0.03 *
Staple food pattern						
Q_1_	1.00		1.00		1.00	
Q_2_	0.92 (0.64, 1.32)	0.64	0.85 (0.58, 1.26)	0.42	0.93 (0.62, 1.38)	0.70
Q_3_	0.67 (0.47, 0.97)	0.03 *	0.76 (0.51, 1.12)	0.16	0.83 (0.56, 1.24)	0.36
Q_4_	0.79 (0.55, 1.13)	0.20	0.85 (0.57, 1.25)	0.40	1.00 (0.67, 1.50)	1.00

Note: Data are presented as OR (95% CI) and are divided into Q1, Q2, Q3, and Q4 groups according to the factor scores of each dietary pattern, with the Q1 group as the reference group; Model 1 was unadjusted; Model 2 was adjusted for gender, age, literacy, and ethnicity; and Model 3 was further adjusted for smoking, alcohol consumption, diabetes mellitus, and CVD based on Model 2. *** *p* < 0.001, ** *p* < 0.01, * *p* < 0.05.

## Data Availability

As the data in this study are part of an ongoing study, the dataset is not readily available. To access the dataset, please contact [Ruli Ma].
